# Perforation couverte du duodénum par arête de poisson - à propos d’un cas

**DOI:** 10.11604/pamj.2017.26.1.9891

**Published:** 2017-01-03

**Authors:** Hajar El Jouadi, Ilyas Derdabi

**Affiliations:** 1 Service de Radiologie, Hôpital Mohammed V, Rabat, Maroc

**Keywords:** Duodénum, perforation, arête de poisson, Duodenum, perforation, fish bone

## Image en médecine

Les perforations de l'estomac et du duodénum secondaire à un corps étranger ont tendance à présenter un tableau clinique plus inoffensif que les perforations situées dans le jéjunum ou l'iléon. Très rarement ces perforations peuvent évoluer spontanément vers la guérison. Nous présentant le cas d'une patiente âgée de 45ans, sans antécédents pathologiques notables admise aux urgences dans un tableau d’appendicite: douleur abdominales localisées au niveau de la fosse iliaque droite (FID) évoluant depuis deux jours, accompagné de quelques épisodes de vomissements, sans autres signes accompagnateurs. L'examen clinique retrouve un patient fébrile à 39°C, stable sur le plan hémodynamique avec à l'examen abdominal une défense abdominale localisée à la FID. Le reste de l'examen clinique est normal par ailleurs. Le bilan biologique montre une hyperleucocytose à 22000 élément/mm^3^ et une CRP à 50. L’échographie abdominale est revenue non concluante. On a complété par un scanner abdominal qui a objectivé un corps étranger radio opaque au niveau de la lumière de D2 (probablement arête de poisson) semblant traverser sa paroi avec micro- bulles de gaz extra luminales et densification de la graisse péritonéale en regard. Le diagnostic de perforation couverte duodénale a été retenu. La patiente a bénéficié d’extraction de corps étranger par endoscopie et mise sous antibiothérapie avec bonne évolution clinique.

**Figure 1:**
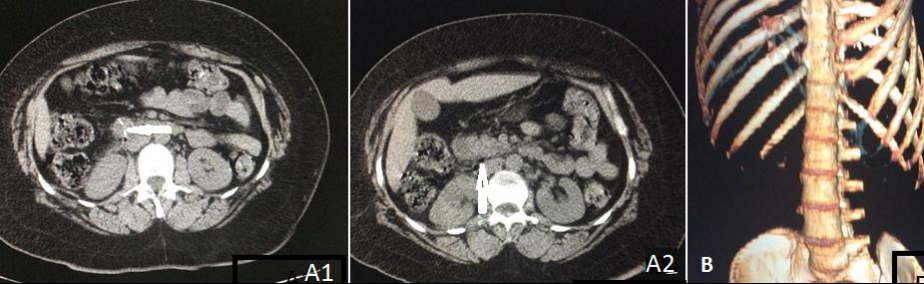
A) TDM abdominal en coupe axiale sans injection de produit de contraste: présence d’une formation linéaire hyperdense au niveau de la lumiere de D2 et traversant sa paroi (A1), micro-bulles de gaz extra luminales et densification de la graisse péritonéale en rétro péritonéal (A2); B) TDM abdominale en reconstruction 3D: montre le corps étranger en forme de V: arête de poisson

